# Organization of control of nosocomial infections in Central Eastern European countries

**DOI:** 10.1007/s10354-018-0671-x

**Published:** 2019-02-06

**Authors:** Franz Allerberger, Bernhard Küenburg

**Affiliations:** 1 Österreichische Agentur für Gesundheit und Ernährungssicherheit (AGES), Spargelfeldstraße 191, 1220 Vienna, Austria; 2 Dr. Ignaz Semmelweis Gesellschaft, Börsegasse 7/4, 1010 Vienna, Austria

Surveillance programs for hospital-acquired infections differ amongst the European states and publicly available information about the methods and indicators of the surveillance systems is frequently lacking. This thematic issue, entitled “Organization of control of nosocomial infections in Central Eastern European countries”, for the first time summarizes and depicts the situation in Central Eastern European countries. A nosocomial infection, also referred to as healthcare-associated infection (HCAI) or hospital infection, is an infection occurring in a patient during the process of care in a hospital or other healthcare facility which was not present or incubating at the time of admission [1]. As is impressively shown in this thematic issue by *Kärki et al.*, of every hundred hospitalized patients, approximately five acquire one of the healthcare-associated infections [2].

In 1985, the U.S. Centers for Disease Control and Prevention’s Study on the efficacy of nosocomial infection control reported that hospitals with four key infection control components—an effective hospital epidemiologist, one infection control practitioner for every 250 beds, active surveillance mechanisms and ongoing control efforts—reduced nosocomial infection rates by approximately one third [3]. The comprehensive WHO Report on the burden of endemic healthcare-associated infection worldwide issued in 2011 highlights the importance of HCAI in different settings and related risk factors, describes advantages and challenges of HCAI surveillance, reports available data on HCAI endemic burden in high-, middle- and low-income countries and their impact worldwide, and finally points out lessons learned and future perspectives [1]. Improving reporting and surveillance systems at the national level; identifying local determinants of the HCAI burden; ensuring minimum requirements in terms of facilities and dedicated resources available for HCAI surveillance at the institutional level, including microbiology laboratories’ capacities; and ensuring that core components for infection control are in place at the national and healthcare setting levels were identified as main solutions and perspectives for improvement [1]. The implementation of surveillance protocols even by itself leads to reduction in infections, a reduction attributed to prompt and timely feedback [4–7]. Although HCAI are largely preventable, the emergence of multidrug-resistant organisms, increasing numbers of immunocompromised patients, and more widely used invasive procedures and medical implantations make prevention challenging. In this thematic issue, *Friedrich A.W.* delineates the differences in the transmission dynamics between microorganisms causing classical infectious diseases and multidrug-resistant microorganisms, underlining the need for an evidence-based approach for today’s infection control strategies.

The drivers for reduction of HACI are multiple and include pressure from patients, regulation by the health authorities, fear of legal action, and convincing guidelines [8]. The cost of HCAIs is another major driver for the reduction of these infections, although in healthcare systems relying on fixed per diem accounting systems, the presence of an HCAI does not necessarily decrease reimbursement revenue for hospitals, as added bed-days can be charged to third-party payers [9]. Countries striving to offer quality care must integrate infection-control improvement initiatives into a wider, comprehensive safety culture. To achieve sustained success in this area, they must harness organizational change to promote, support, and reinforce infection prevention and control activities. We need to learn from each other in order to facilitate optimal infection control.

Recently published essential structural components and indicators of infection prevention and control programs stress the need for comprehensive approaches that integrate multimodal and multidisciplinary solutions and strive to reinforce an organizational culture of patient safety [10]. As evidenced by the 18 manuscripts from CEE countries [11], tremendous differences exist concerning legal basis, structural components and indicators of infection prevention and control approaches in those states. However, the heterogeneity of structural factors makes it important to avoid any temptation to simply “transplant” interventions from one country to another without a robust understanding of contextual factors surrounding the interventions and the characteristics of the host setting [12].

Modern infection control is grounded in the work of Ignaz Semmelweis, who in the 1840s demonstrated the importance of hand hygiene for controlling transmission of infection in hospitals. The Semmelweis Foundation, which initiated this thematic issue, strives to increase the hygiene level in healthcare institutions in order to save lives by increasing the public awareness and creating political momentum through information and initiatives on different levels. The biannual Semmelweis Congress—rotationally tacking place in Vienna or Budapest—especially aims to foster the improvement of the control of nosocomial infections in Central Eastern European countries. The fact that all 18 states invited for articles on their national policies on HCAI control actually delivered manuscripts for this thematic issue, surely augurs well for future infection control and prevention programs central to protecting patients from the harm of nosocomial infections.
